# A critical perspective on Markov state model treatments of protein–protein association using coarse-grained simulations

**DOI:** 10.1063/5.0039144

**Published:** 2021-02-22

**Authors:** Ziwei He, Fabian Paul, Benoît Roux

**Affiliations:** 1 Department of Chemistry, The University of Chicago, 5735 S Ellis Ave., Chicago, Illinois 60637, USA; 2 Department of Biochemistry and Molecular Biology, The University of Chicago, 929 E. 57th Street W225, Chicago, Illinois 60637, USA

## Abstract

Atomic-level information is essential to explain the specific interactions governing protein–protein recognition in terms of structure and dynamics. Of particular interest is a characterization of the time-dependent kinetic aspects of protein–protein association and dissociation. A powerful framework to characterize the dynamics of complex molecular systems is provided by Markov State Models (MSMs). The central idea is to construct a reduced stochastic model of the full system by defining a set of conformational featured microstates and determining the matrix of transition probabilities between them. While a MSM framework can sometimes be very effective, different combinations of input featurization and simulation methods can significantly affect the robustness and the quality of the information generated from MSMs in the context of protein association. Here, a systematic examination of a variety of MSMs methodologies is undertaken to clarify these issues. To circumvent the uncertainties caused by sampling issues, we use a simplified coarse-grained model of the barnase–barstar protein complex. A sensitivity analysis is proposed to identify the microstates of an MSM that contribute most to the error in conjunction with the transition-based reweighting analysis method for a more efficient and accurate MSM construction.

## I. INTRODUCTION

One of the most important questions in biology is how living cells communicate and respond to the flow of information at the molecule level. To decipher the molecular basis of cellular communication, one must explain the specific interactions governing protein–protein recognition in terms of structure and dynamics.^1–3^ While the protein–protein equilibrium binding affinity and specificity are certainly important, a characterization of the kinetic aspects of association and dissociation is perhaps of even greater significance to understand the time-course of biological processes.^4^ In principle, atomic-level information is essential to study protein complexes in terms of structure and dynamics. However, the long timescales and high dimensionality present outstanding computational challenges in computer simulations of rare events.^5^

Markov state models (MSMs) provide a powerful framework for characterizing the kinetics of complex molecular systems.^6–11^ MSMs are discrete state and discrete time stochastic master equation models. Building an MSM involves defining a set of discrete microstates within a subspace of collective variables (features), and then estimating the hopping transition probabilities between such states at a fixed lag-time interval from the information generated by detailed dynamical simulations.^12^ Assuming that the resulting MSM thus constructed is indeed representative of the system of interest, the framework can then be used as a generator to predict any equilibrium or long-term kinetic properties at low computational cost.

The overall accuracy and usefulness of MSM analysis is typically burdened by two opposing problems. The first problem arises from the need to achieve Markovian dynamics. An MSM must satisfy ergodicity, but often important transitions are not sampled due to computational limits and are missing in the MSM. In addition, the evolution of the system monitored at a given lag-time interval exhibits non-Markovian correlated dynamics when the trajectories are mapped onto a set of coarsely defined microstates. Immediate remedies to reduce such non-Markovian effects are to choose a longer lag-time or refine the definition of the microstates by using a featurization space of higher dimensionality with more collective variables. However, as the lag-time and the number of microstates are increased, the finite amount of information from the detailed simulations becomes rapidly insufficient to determine the larger number of transition probabilities accurately. Thus, as we try to address the first problem, achieving Markovian dynamics, the statistical accuracy of the MSM breaks down, causing the second problem.

Different strategies have been devised to mitigate these two contradictory problems by trying to efficiently identify the smallest number of most relevant features expected to display the least amount of non-Markovian dynamics. One such method is the time-lagged independent component analysis (TICA),^13,14^ which was proposed to process high-dimensional data without the loss of kinetically relevant information. Unfortunately, the optimal selection of input features and the process of discretization to define the microstates are often unclear, and there are various different ways one can construct an MSM for the same system.^6,7^ Furthermore, the accuracy of the MSM relies quite heavily on having a well-sampled configurational space. Despite the recent advances in computational modeling and achievements in MSMs applied to large biomolecular systems,^15,16^ the construction of robust and well converged MSM from full atomistic simulations still remains a highly demanding feat. By itself, the MSM framework does not directly help improve the exploration of rare events and (high free energy) configurations. Sampling issues must be tackled indirectly through sensitivity analysis and adaptive strategies. Different methodological aspects may be brought to bear on the problem to ensure an optimal outcome, including featurization, enhanced sampling techniques,^16,17^ and sensitivity analysis^18–21^

Our principal goal is to test the robustness and reliability of the methodology by exploring different strategies for the efficient construction of MSMs. To maintain complete control over the convergence of the present analysis and circumvent the statistical uncertainties caused by sampling issues, a simplified coarse-grained (CG) model of the barnase–barstar protein complex was used for all the simulations. Several MSMs were built from different sets of features as well as combinations of biased and unbiased simulations to understand how these inputs may affect the resultant thermodynamic and kinetic observables. We then re-examine the transition-based reweighting analysis method (TRAM).^17^ Using an approach similar to the eigenvalue-based sampling,^22–24^ we propose a sensitivity analysis^18–21^ that can identify regions of undersampling and efficiently add in biased simulations only where necessary.

## II. METHODS

### A. Coarse-grained system

**FIG. 1.**
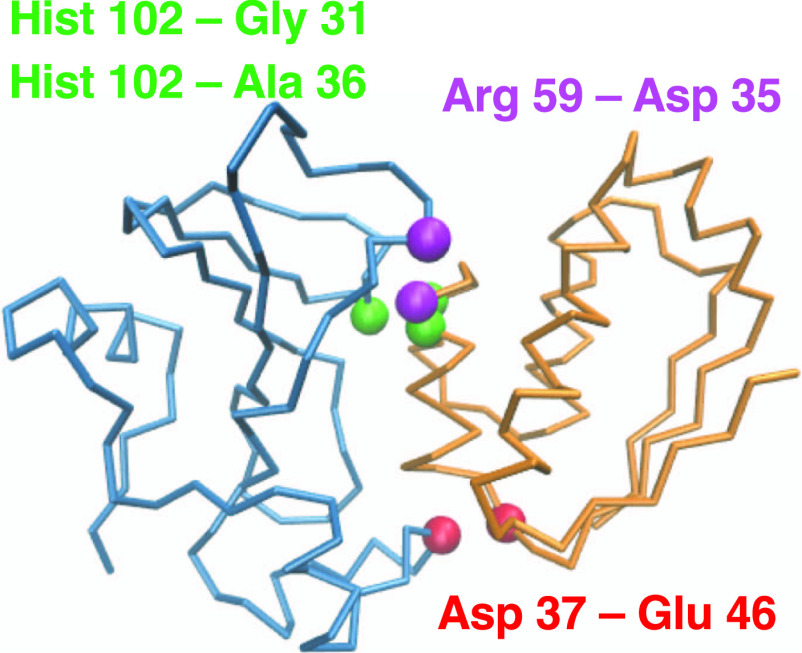
The barnase–barstar protein complex with one coarse-grained (CG) particle per residue. In order to model the interactions in the simplified protein complex, four Lennard-Jones potentials between key non-bonded particles, corresponding to the important residues involved in binding, were chosen to simulate association in the correct native bound state. The key pairwise residues are depicted as CG beads.

The barnase–barstar system is taken from the crystal structure 1BRS Protein Data Base ID.^25^ Chain B is selected for barnase and chain D for barstar. The CG representation is constructed by mapping each amino acid residue as a single bead with its mass and position corresponding to the C*α*. We designed our CG potential as a Gō-like model^26,27^ using attractive potentials to represent the pairwise contacts of the native complex. Four Lennard-Jones (LJ) 6-12 potentials with a well depth of 3.0 kcal/mol were introduced between the non-bonded beads of barnase and barstar (Fig. 1) to accurately simulate the association in the native bound state. The four Gō-like contact pairs, listed in Table I, were selected due to their importance for binding as reported in the literature.^28–30^

**TABLE I.** Gō-like contact pairs chosen from key amino acid residue pairs on barnase and barstar with minimum distances *R*_min_.

Barnase	Barstar	*R*_min_ (Å)
Asp 37	Glu 46	4.96
Arg 59	Asp 35	5.65
Hist 102	Gly 31	4.82
Hist 102	Ala 36	5.83

In addition, root-mean-square deviation (RMSD) restraints were applied to each protein to maintain their folded conformation. The complex was enclosed in a finite spherical volume with a radius of 73.46 Å using a flat-bottom potential, yielding an effective concentration of 1 *μ*M. The barnase was restrained at the origin in position and orientation, while barstar was allowed to freely diffuse in the cavity. Because the protein complex is invariant by translation and rotation, this does not affect the equilibrium features of the system. 25 independent, unbiased Langevin molecular dynamic (MD) simulations were performed at 300 K with a damping constant of 1 ps^−1^ and time step of 1 fs for a total aggregate simulation time of 25 *µ*s. Umbrella sampling (US) simulations were carried out using harmonic biasing potentials chosen along the distance between the center-of-mass (COM) of barnase and the COM of barstar, with spring constants of 1 kcal/mol. 70 windows were assigned along the reaction coordinate from 4 Å to 73 Å at 1 Å intervals, yielding 200 ns of simulation time per window at a 5 fs time step. All simulations were performed using the NAMD program^31^ with the CHARMM^32^ force field parameter file to choose non-bonded pairs for implementing Gō contacts. The visualization program VMD^33^ was used to render the barnase–barstar complex.

### B. Markov state model construction

We construct four MSMs using different combinations of biased and unbiased trajectories and different choices in featurization. These selections account for the limitations MSMs often face due to (1) undersampling and (2) non-optimal selection of features due to the many possible reaction pathways that arise from the simulation of huge biomolecules. We evaluate the performances of the MSMs by comparing them with the properties calculated from the raw trajectories of the MD and US simulations. The MSMs and their construction input parameters are summarized in Table II. Detailed discussions of the models follow in Sec. III.

**TABLE II.** MSMs constructed from different featurization and simulation methods. Models were estimated at a lag time of 12 ns.

Model name	Features	Simulation	Microstates
MSM-6D	Four TICA components from long-lived pairwise contacts,	MD	550
	RMSD with respect to the native bound state, and COM distances		
TRAM-6D	Four TICA components from long-lived pairwise contacts,	MD + US	100
	RMSD with respect to the native bound state, and COM distances		
TRAM-1D	COM distances	MD + US	100
TRAM-1D-inv	Squared inverse COM distance	MD + US	100

MSM-6D is a traditional MSM built from unbiased MD trajectories using six features corresponding to the four slowest linear combinations of pairwise contacts computed from TICA, the minimum RMSD of the complex with respect to the native bound state, and the COM distances between the barnase protein and the barstar protein. We will treat MSM-6D as the reference MSM since it is a traditionally constructed MSM using only unbiased simulations and has a relatively long aggregate simulation time of 25 *µ*s with many observed association and dissociation events. TRAM-6D was built using the same approach as MSM-6D with the addition of biased US trajectories. TRAM-1D was generated using only the COM protein distances from the MD and US trajectories. Finally, TRAM-1D-inv was built upon the squared inverse COM distances as features to consider an indirect reaction coordinate. Clustering was performed using k-means,^34,35^ and the number of microstates was determined by the elbow method,^36^ which optimizes the minimization of the intra-cluster variance. The models were constructed with lag times of 12 ns. The MDTraj^37^ software was used for trajectory analysis. The PyEMMA^38^ software and a few functionalities of the msmtools package were used to construct the MSMs and perform several analyses. Figures were rendered using Matplotlib.^39^

For MSM-6D and TRAM-6D, which rely on a larger set of features, a number of methods were employed to make the MSM construction feasible despite the large volume of data. Given the 110 residue beads on barnase and 89 on barstar, we would have to work with 110 × 89 = 9790 pairwise distances. In order to reduce the computational effort in such a high dimension, we considered only the pairwise distances that were deemed kinetically relevant. Employing a similar strategy used by Plattner *et al.* with their hidden MSM built upon the full atomistic simulations of the barnase–barstar complex,^15^ we obtained 817 long-lived pairwise contacts that were within a distance of 12 Å and bound for at least 1 ns.

TICA was utilized to further reduce dimensionality. TICA is a powerful dimensionality reduction algorithm that extracts the most kinetically relevant linear combinations of the long-lived pairwise contact distances. Briefly, TICA first computes the time-lagged covariance matrices **C**(τ) from a given set of mean-free input data *r*(*t*) (e.g., the long-lived pairwise distances) at time *t* with the following elements:

$$c_{ij}\left(\tau \right)=\left\langle r_{i}\left(t\right)r_{j}\left(t+\tau \right)\right\rangle$$ (1)

$$=\frac{1}{N-\tau -1}\overset{N-\tau }{\underset{t=1}{\sum }}r_{i}\left(t\right)r_{j}\left(t+\tau \right),$$ (2)

where τ is the lag time and *N* is the size of the data. Then, solving for the generalized eigenvalue problem gives

C(τ)U=C(0)UΛ, (3)

where **U** is an eigenvector matrix consisting of time-lagged independent components (ICs) as the columns and **Λ** is a diagonal eigenvalue matrix. The dataset **r**(*t*) is then projected onto the TICA space that maximizes the autocorrelation of the transformed coordinates,

$$z^{⊤}\left(t\right)=r^{⊤}\left(t\right)U.$$ (4)

We reduced down to the desired number of dimensions by choosing a subspace of only the first few columns of **U**. A more detailed discussion of TICA can be found in Refs. 13 and 14.

For this study, we kept the four slowest ICs from TICA that had noticeably slower timescales compared to the rest of the ICs, as demonstrated in Fig. S1 of the supplementary material. Then, the RMSD and COM distances were added as additional features to include physical observables that are more intuitively understandable. Clustering was performed on this six-dimensional feature space. This feature selection is similar to the hidden MSM of the all-atom barnase–barstar reported by Plattner *et al.*^15^ Hence, we can avoid any uncertainty underlying the number of dimensions during the MSM construction by employing a similar feature selection for the CG descriptions (MSM-6D and TRAM-6D) that is based on the atomistic MSM. We note that selecting the six features and other MSM hyperparameters for MSM-6D and TRAM-6D manually was straightforward for the present CG complex. However, for more complicated systems, the Generalized Matrix Rayleigh Quotient (GMRQ)^40–43^ and the variational approach for Markov processes (VAMP) score^44,45^ would provide useful tools for systematically determining the optimal features and MSM hyperparameters.

### C. Determination of the optimal cutoff distance

For the purpose of comparing the kinetic rates and binding constants of the MSMs with those calculated from the raw analyses of the trajectories, we first have to choose a cutoff distance that clearly delineates between the bound and unbound states in the simulations. For *r*_cut_ values that are much smaller or larger than an optimal *r*_cut_, there will be faster fluctuations and numerous rapid recrossings on a short timescale. We want to determine the *r*_cut_ for which the influence of such rapid fluctuations is minimized. Here, for *r*_cut_ in the range of 25 Å–35 Å along the COM distance, an indicator state function *h*(*t*) was assigned to be equal to 0 when unbound and 1 when bound,

$$h\left(t\right)=\left\{\begin{array}{cc} 0\; & ifunbound,\\ 1\; & ifbound. \end{array}\right.$$ (5)

**FIG. 2.**
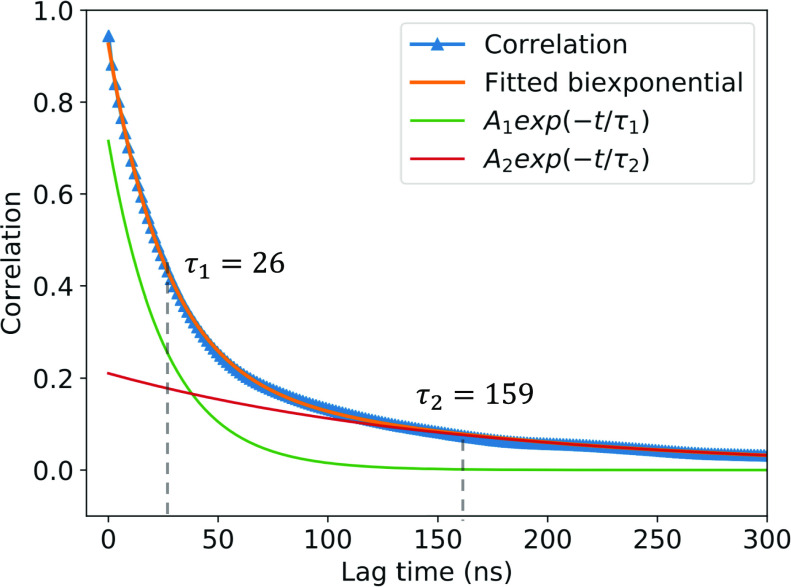
Autocorrelation of the bound state indicator (blue triangles) for *r*_cut_ = 29 Å. A biexponential decay curve was fitted (orange) to the autocorrelation using two exponential functions $A_{1}e^{-t/\tau_{1}}$ and $A_{2}e^{-t/\tau_{2}}$ (red and green, respectively), where *A*_1_ = 0.715, τ_1_ = 26.1 ns, *A*_2_ = 0.211, and τ_2_ = 159.4 ns.

Then, the time-correlation function, averaged from the aggregate trajectories, was calculated for each *r*_cut_ value,

C=⟨h(0)h(τ)⟩, (6)

where τ is the lag time. Normalizing the time-correlation function, we can rewrite Eq. (6) as

$$C=\frac{\sum_{i=1}^{N-\tau }\left(h_{i}-\tilde{h}\right)\left(h_{i+\tau }-\tilde{h}\right)}{\sum_{i=1}^{N}{\left(h_{i}-\tilde{h}\right)}^{2}},$$ (7)

where *N* is the total simulation time length and $\tilde{h}$ is the averaged data. In order to determine the relaxation lag time, the correlations were fitted (Fig. 2) using biexponential decaying functions of the form

$$A\left(t\right)=A_{1}e^{-t/\tau_{1}}+A_{2}e^{-t/\tau_{2}}.$$ (8)

Upon fitting, the *r*_cut_ values were adjusted until the relaxation time in the correlation function was the longest (i.e., when the biexponential τ’s were the longest).

**FIG. 3.**
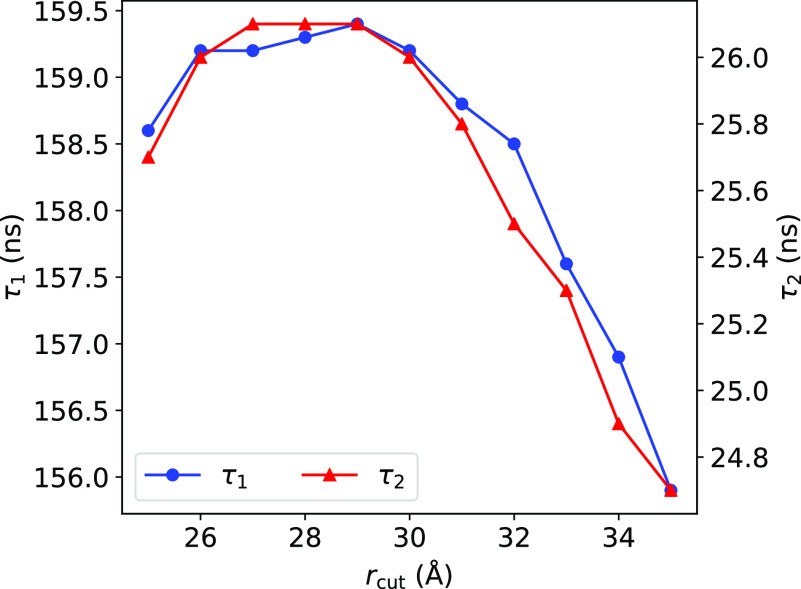
Relaxation times as a function of *r*_cut_. The lag times τ_1_ and τ_2_ peak at an *r*_cut_ of 29 Å, and we choose this *r*_cut_ value as the bound state cutoff to minimize any statistical fluctuations in our trajectory analyses.

As demonstrated in Fig. 3, the optimal value of *r*_cut_ yielding the largest relaxation times is 29 Å, although we note that the results are fairly similar for a COM distance varying between 25 Å and 35 Å (supplementary material Table S1 lists the *r*_cut_ values and the biexponential lag times, τ_1_ and τ_2_). Accordingly, we define the bound and unbound states as

$$state=\left\{\begin{array}{cc} bound\; & \text{if}r\leq r_{cut},\\ unbound\; & \text{if}r>r_{cut}, \end{array}\right.$$ (9)

where a bound state is defined as having a COM distance between barnase and barstar that is within 29 Å and an unbound state is defined as having a COM distance greater than 29 Å. Since the complex has been reported to go through a loosely bound state before reaching the final native bound state,^15,46^ a τ_1_ of 26 ns can be thought of as the timescale to achieve the loosely bound state, while a τ_2_ of 159 ns can be taken as the average lifetime of the fully bound state. This *r*_cut_ value was employed for analysis of the MD and US trajectories in Table III of Sec. III.

**TABLE III.** Equilibrium binding constants and kinetic rates obtained from the five different MSMs.

Model name	$K_{eq}^{PMF}\;\left(\times 10^{5}{Å}^{3}\right)$	$K_{eq}^{Pbound}\;\left(\times 10^{5}{Å}^{3}\right)$	$k_{on}\;\left(\times 10^{13}{Å}^{3}s^{-1}\right)$	*k*_off_ (×10^7^ s^−1^)
MD	9.24	8.86	2.67	2.98
US	9.04	…	…	…
MSM-6D	…	8.83 ± 0.17	2.31 ± 0.04	2.70 ± 0.03
TRAM-6D	…	8.72 ± 0.17	2.37 ± 0.04	2.75 ± 0.03
MSM-1D	…	8.72 ± 0.20	2.50 ± 0.03	2.86 ± 0.04
TRAM-1D	…	8.67 ± 0.16	2.44 ± 0.03	2.89 ± 0.04
TRAM-1D-inv	…	8.66 ± 0.20	2.43 ± 0.04	2.88 ± 0.04

### D. Calculation of thermodynamic and kinetic properties from MSMs

In order to understand protein–protein interactions by means of MSMs, we must be able to calculate thermodynamic and kinetic quantities from them. The equilibrium binding constant and the Δ*G*_b_ of binding can be obtained from the stationary distribution, *π*, which gives the equilibrium probability distribution by the first left eigenvector from the MSM transition matrix, **T**, as follows:

π=π T. (10)

We can then calculate the binding constant from the ratio of the probabilities of the bound to unbound states given by *π*,

$$K_{eq}C=\frac{P_{bound}}{P_{unbound}},$$ (11)

where *C* = *V*^−1^ is the concentration and *V* is the volume corresponding to the “bulk” region,

$$V=\frac{4}{3}\pi \left(r_{cavity}^{3}-r_{cut}^{3}\right).$$ (12)

An *r*_cavity_ equal to 73.46 Å is the radius of the spherical cavity used to enclose the protein complex during simulation and *r*_cut_ equal to 29 Å represents the cutoff distance used to delineate between the bound and unbound states. The volume of the bulk region yields a concentration of 1 *μ*M. The binding free energy can be defined as

$$\Delta G_{b}\;:=\;-k_{B}T\;\ln\left(\frac{P_{bound}}{P_{unbound}}\right).$$ (13)

The binding constant can also be calculated by integrating over the radial potential of mean force (PMF),

$$K_{eq}=\int_{0}^{r_{cut}}dr\;4\pi r^{2}\;e^{-\beta W\left(r\right)},$$ (14)

where *β* = 1/*k*_B_*T*, *k*_B_*T* = 0.596 kcal/mol. Here, it is assumed that the PMF has been offset to have lim_*r*→∞_*W*(*r*) = 0.

Perron-cluster cluster analysis (PCCA) is a method that clusters eigenvectors in order to define metastable, or long-lived, states in an MSM.^47,48^ We performed the PCCA++ method implemented in PyEMMA to define two metastable states, the bound state and the unbound state, in order to obtain the mean first passage times (MFPT) between these two states. Kinetic rates of association and dissociation can be calculated accordingly,

$$k_{on}=\frac{1}{{MFPT}_{on}\;C},$$ (15)

$$k_{o\mathrm{ff}}=\frac{1}{{MFPT}_{o\mathrm{ff}}}.$$ (16)

## III. RESULTS

**FIG. 4.**
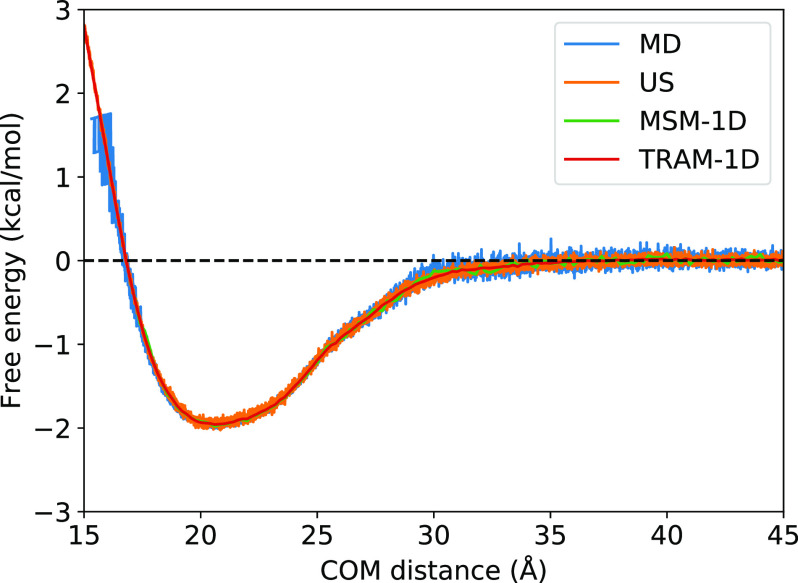
Free energy profiles of the raw trajectories, MSM, and TRAM. The free energy profiles obtained from a raw MD trajectory of 25 *µ*s aggregate simulation time (blue), the PMF of a US simulation of 200 ns per window (orange), a one-dimensional traditional MSM from COM distance feature and 25 *µ*s aggregate simulation time (green), and TRAM-1D (red) show near identical agreement, proving the robustness of the MSM methodology even when using only one feature.

In this section, we now discuss the results obtained from the four MSMs described in Table II. Figure 4 compares the free energy profiles of a one-dimensional MSM (25 *µ*s MD simulation) and TRAM-1D (25 *µ*s MD simulation and 200 ns per window US simulation) with the PMFs from the raw MD and US trajectories. The free energies, MFPTs, and resultant binding constants and rates are within agreement, indicating that using just the one-dimensional COM distance can adequately capture the binding process of this protein complex. Table III summarizes the thermodynamic and kinetic properties calculated from the MSMs. The kinetic rates and binding constants were calculated from the MSMs and demonstrate that the MSM methodology is robust and consistent. Notably, even stripping the features down to only one dimension seems to produce MSMs that can recapitulate the thermodynamics and kinetics.

Calculations were also performed for the raw unbiased (MD) and biased (US) simulations for comparison with the MSMs. The binding constants obtained by integrating the free energy profiles from 0 to *r*_*cut*_ [Eq. (14)] are listed under $K_{eq}^{PMF}$, and the binding constants obtained by calculating the probability ratios of bound and unbound states [Eq. (11)] are listed under $K_{eq}^{Pbound}$. The association and dissociation rate constants are listed under *k*_on_ and *k*_off_, respectively. We will describe each of the MSMs in more detail in Secs. III A and III B.

### A. MSM-6D

**FIG. 5.**
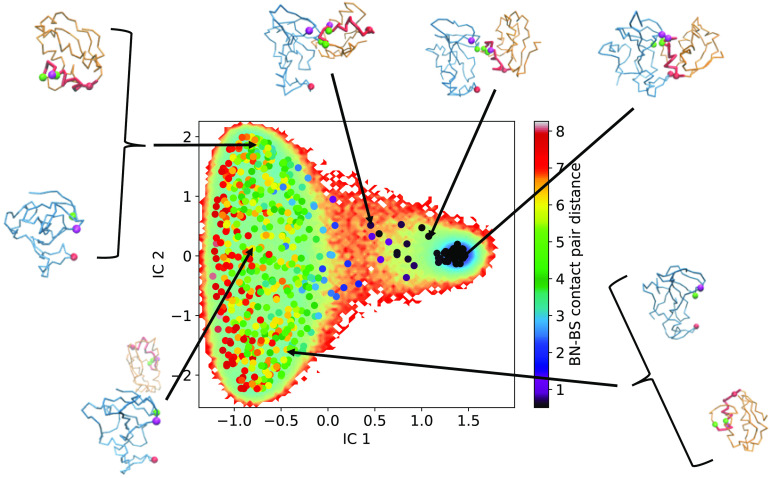
Representative structures of the MSM-6D microstates. The centroids from k-means clustering are colored by the COM distance of the complex and plotted on top of the free energy landscape along the two slowest modes of TICA. Representative structures are taken from each of the centroids to give a better idea of the microstate assignment. Starting from the top right and going counterclockwise, we have clusters representing the bound state, an intermediate state where one LJ pair is separated, an intermediate state where two LJ pairs are separated, and three dissociated states.

We start off by examining MSM-6D, which is a conventional MSM using a set of high-dimensional features from unbiased trajectories. As illustrated in Fig. 5, MSM-6D can clearly distinguish the bound and unbound configurations. The free energy landscape along the two slowest TICA components, or independent components (IC) 1 and 2, reveal a free energy well corresponding to the bound configurations and another well for completely dissociated structures. The 550 centroids in Fig. 5 are colored based on the COM distance between barnase and barstar.

The first IC corresponding to the slowest degree of freedom represents the binding pathway along the COM distance, while the second, third, and fourth ICs can be interpreted as the orientational changes during association and dissociation. Although the free energy landscape along ICs 2, 3, and 4 does not change as drastically as that for IC 1, these slow modes are still important for association. Since the rate-limiting step involves a partially bonded conformation where some of the pairwise contacts are formed while others are not, the orientation of the proteins may change even though the COM distance between them remains the same. In other words, by including the four ICs from TICA, our six-dimensional MSM effectively encodes both translational and orientational contributions in the course of protein association. The full relationship among the four ICs is depicted in Fig. S2 of the supplementary material.

**FIG. 6.**
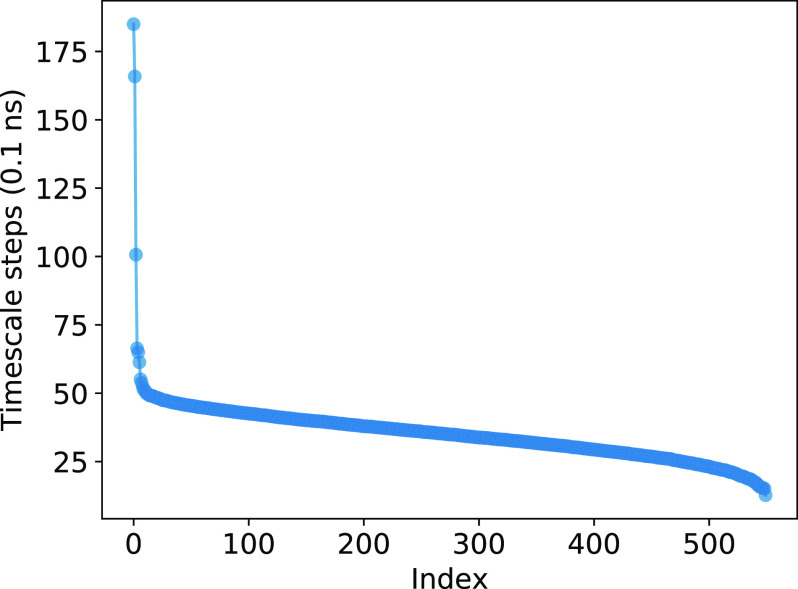
Timescales of MSM-6D with 550 microstates. There are two timescales lasting longer than 15 ns and one lasting around 10 ns, indicating that there should be three or more long-lived, or metastable, states.

**FIG. 7.**
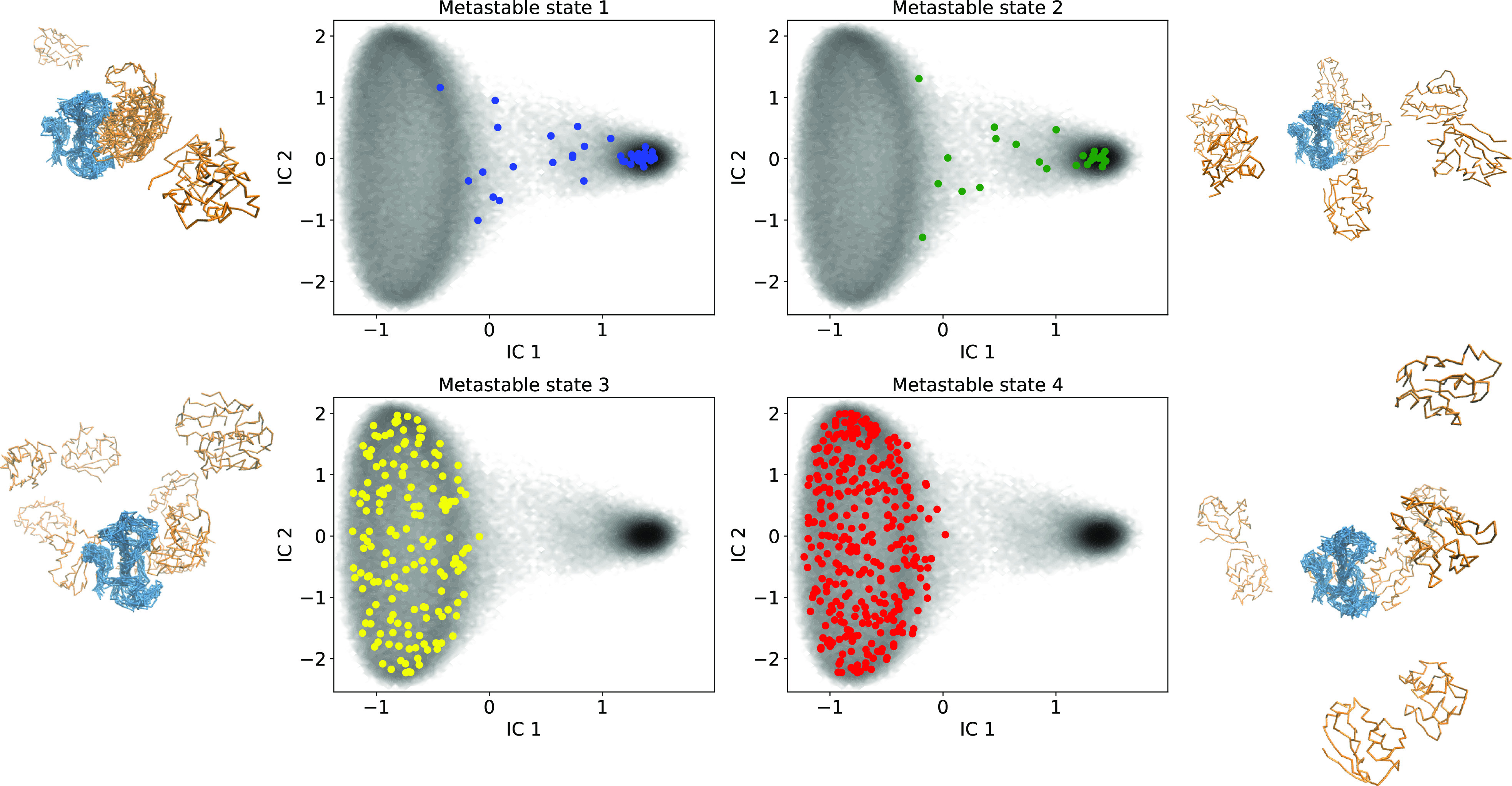
Four metastable states of MSM-6D determined by PCCA. Metastable state 1 consists of primarily bound states. Metastable state 2 consists of intermediate or loosely bound states. Metastable states 3 and 4 consist of completely dissociated complexes. PCCA assignments use fuzzy clustering, while a crisp assignment is employed to visualize how the 550 microstates map to each of the metastable states. The gray shading is the same free energy landscape shown in Fig. 5.

The MSM-6D timescales plotted in Fig. 6 indicate two much slower timescales at 17 ns and 19 ns and another relatively slower timescale at 10 ns, suggesting that there should be at least three or four important metastable states. Then, carrying out PCCA with four states, as illustrated in Fig. 7, we generated one metastable state where the complex is primarily in the bound state, a metastable state consisting of intermediate states and loosely bound states, and two metastable states with where the complex is completely dissociated. The dissociated conformations in metastable states 3 and 4 are distinguished by the orientational position of barstar with respect to barnase. It should be noted that the PCCA metastable assignment uses fuzzy clustering. For example, metastable state 1 corresponding to the bound state still contains a few structures that clearly correspond to dissociated states. We can perform a crisper assignment by manually selecting the set of states, which is fairly straightforward, but we choose PCCA here because it is more generally applicable for systems with reaction coordinates that are not so readily straightforward. For the purpose of obtaining the MFPTs of the bound and unbound states in order to calculate the kinetic rates listed in Table III, we generated a two-state PCCA.

### B. TRAM

TRAM allows the estimation of MSMs by stitching together the different thermodynamic and kinetic information from the biased and unbiased simulations.^48^ As we ultimately want to take advantage of both biased and unbiased data, in Secs. III B 1 and III B 2, we examine MSMs built from biased trajectories in addition to unbiased trajectories. In particular, we show that incorporating a sensitivity analysis into the TRAM construction can efficiently improve results.

#### 1. TRAM-1D and TRAM-1D-inv

In this section, we present two interesting cases that test the usefulness and robustness of TRAM. For featurization, we consider only the one-dimensional COM distance between barnase and barstar in order to understand how a minimum set of features would compare with higher dimensional models.

**FIG. 8.**
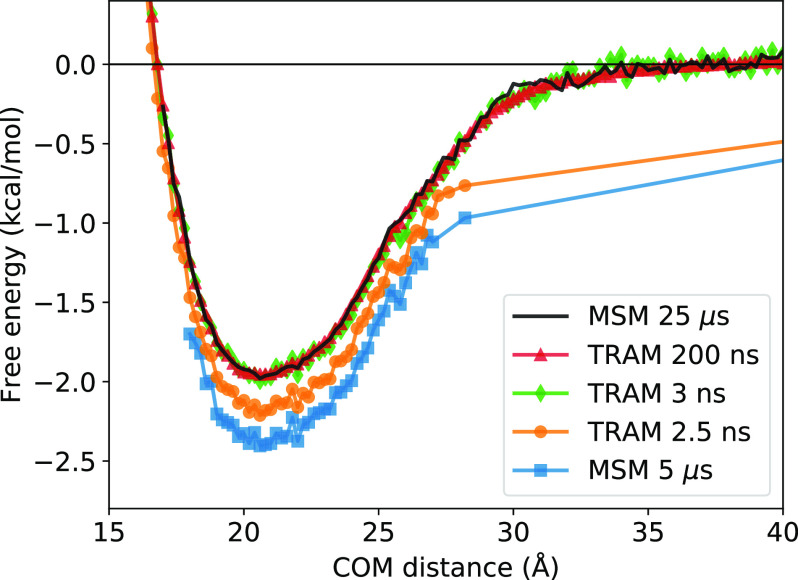
Efficiency of TRAM. The MSM from short MD trajectories of only 5 *µ*s of simulation time (blue square) does not recover as agreeable of a free energy profile as the TRAM models constructed from (a) 5 *µ*s of MD simulation and 2.5 ns per window US simulation (orange dot), (b) 3 ns per window US simulation (green diamond), and (c) 200 ns per window US simulation (red triangle, TRAM-1D in Table III). With the addition of only 3 ns of US simulation, TRAM is within near agreement to the reference MSM estimated from a long MD simulation time of 25 *µ*s (black).

**FIG. 9.**
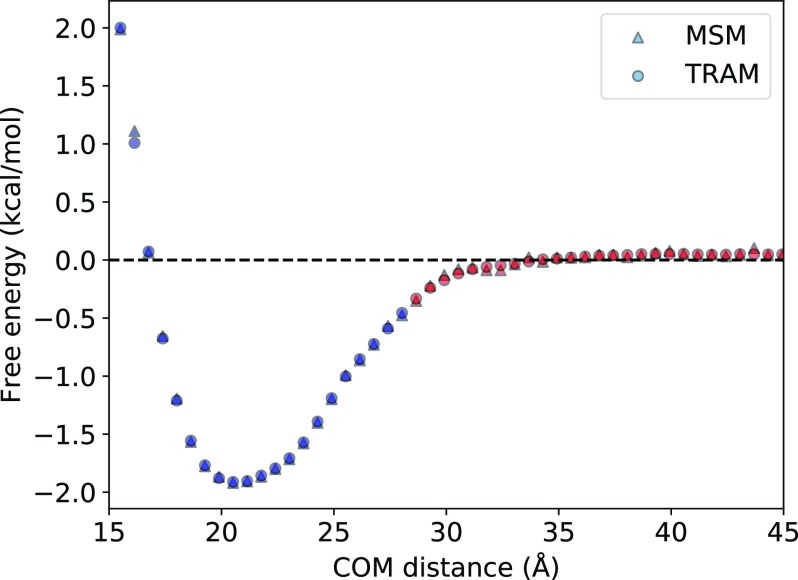
PCCA metastable states of MSM-1D and TRAM-1D. The bound (blue) and unbound (red) states are able to be clearly distinguished when using COM distances as the only feature.

Given an undersampled and relatively short 5 *µ*s MD trajectory, the MSM built from it using a one dimensional feature space, in this case the COM, is quite inaccurate, as shown by the MSM free energy profile in Fig. 8. However, adding minimal biased US data to an MSM built from undersampled MD simulation can recapitulate the same free energy profiles as an MSM from long simulations of 25 *µ*s. The addition of only 3 ns (per window) of US simulation drastically improves upon the MSM via reweighting of the thermodynamics with TRAM. Compared to the MSM-6D from very long simulations, the MSM from short simulations yields a binding constant and rates that are vastly different. The addition of biased trajectories by way of TRAM recuperates the thermodynamics and kinetics seen from MSM-6D. The TRAM-1D model in Table III corresponds to TRAM model with 200 *µ*s of biased simulations in Fig. 8. Figure 9 demonstrates that PCCA assignment is able to clearly distinguish between the bound and unbound states along this feature.

**FIG. 10.**
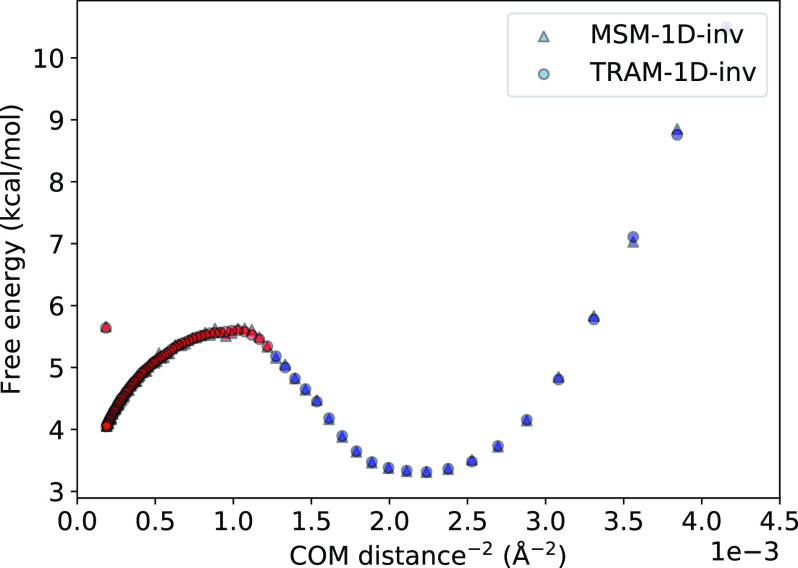
PCCA metastable sets generated from MSM and TRAM using 1/COM^2^ as the feature. The bound (blue) and unbound (red) states are still able to be clearly distinguished when using this indirect feature.

Even though the bound and unbound states can be straightforwardly distinguished by the COM distances in the present model, large biomolecules often exhibit ambiguous binding pathways that involve various internal degrees of freedom, and determining a correct reaction coordinate may be highly challenging. Still, a desired MSM model should be able to extract important binding properties using indirect reaction coordinates. We choose the squared inverse COM distances as features for TRAM-1D-inv to examine how well it can still capture the binding process and whether this less direct feature may introduce any instability near the short-range binding distance, compared with the direct COM distances. Remarkably, Fig. 10 shows that the free energy profile of TRAM-1D-inv is in near identical agreement with the unbiased MSM, and the thermodynamic and kinetic properties are also well-reproduced. Even though it is not a direct reaction coordinate of the binding pathway, this type of feature can resolve the bound and unbound states. Error estimation was performed using bootstrapping by sampling blocks of trajectories, but the error bars of the PMFs in Figs. 9 and 10 are three orders of magnitudes smaller and, thus, too small to be visualized.

It is interesting to note that these one-dimensional models are able to faithfully recapitulate the thermodynamics and kinetics just as well as the more computationally expensive models, MSM-6D and TRAM-6D. This can be understood from the timescales of TICA shown in Fig. S1, where the first timescale is predominately larger than the other timescales by an order of magnitude. This suggests that the COM distance is the most crucial collective variable for understanding the current system, even though some orientation and internal motions are also present and should be important in order to fully understand the overall processes. Therefore, from these one-dimensional models, we conclude that the binding pathway may be accurately described using only a few well-chosen collective variables, which could prove to be extremely useful for MSMs of large protein complexes in which the binding pathway is not completely unambiguous. Future work may involve examining such MSMs by employing features such as the dihedral angles of residues that are not explicitly related to the COM distances or any other inherently intuitive distance criteria for the binding process.

#### 2. TRAM-6D: Optimizing efficiency with biased data

Since a major challenge in MD studies is to obtain enough sampling, we often end up with undersampled unbiased data that have high statistical uncertainty. Earlier work involving eigenvalue-based sampling have attempted to improve the accuracy of MSMs by starting simulations from the states that present the most uncertainties in their eigenvalues or kinetics. While a few error analysis methods have been proposed in literature for use with adaptive sampling,^22^ here we apply a simple sensitivity analysis in order to pinpoint the discretized microstates of an MSM that contribute the most error. Then, the problematic microstates are mapped back to their corresponding subset of features (i.e., COM distance between the proteins) in order to add biased simulations for TRAM only where the additional windows will provide the most benefit. In this section, we will demonstrate that this idea is rather straightforward and easy to implement.

**FIG. 11.**
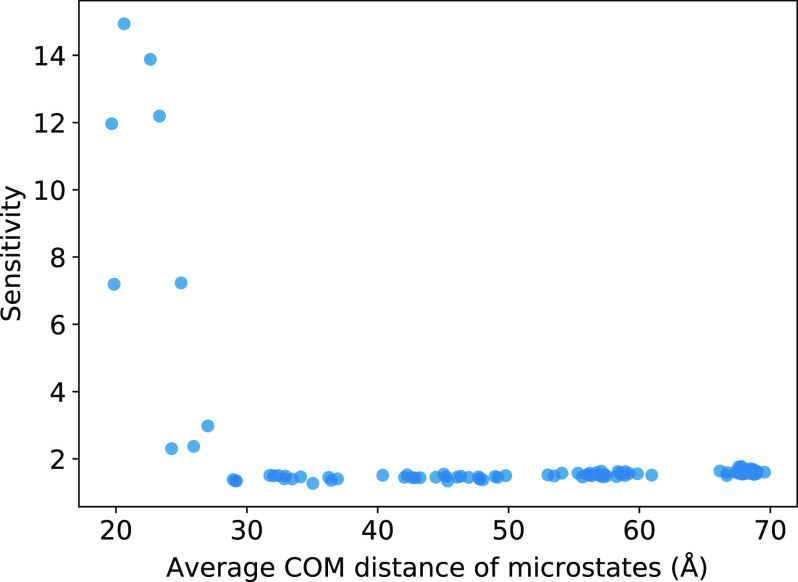
The *π* global sensitivity as a function of the average COM of each MSM microstate. Higher *π* sensitivities correspond to the microstates with shorter averaged COM distances that belong to the bound or intermediate states.

The relationship between the sensitivity and the complex configuration is depicted in Fig. 11. The highly sensitive microstates correspond to the bound states and the loosely bound intermediate states, which is expected since these states are the key players in the binding process. We note that while this may seem readily apparent here for our CG system, it may not be as straightforward for larger and more complex biomolecular systems that have multiple different binding pathways. For such highly complicated cases, the sensitivity analysis can be even more advantageous for pinpointing regions of undersampling.

To employ this sensitivity analysis approach, we first construct a traditional MSM. A local sensitivity matrix can be computed for each element from the transition matrix,

$$S_{ij}=\left(\frac{\partial f\left(T\right)}{\partial T_{ij}}\right),$$ (17)

where *f*(**T**) is the observable of interest and **T** is the MSM transition matrix.^18,19^ In the present case, the observable of interest is the equilibrium distribution of the states *π*_*i*_(**T**) defined by the transition matrix of the MSM. A variance-based sensitivity analysis, also known as the Sobol method,^20,49^ was used to obtain the global sensitivities as follows:

$$S_{global}=\underset{ijkl}{\sum }\;S_{ji}\text{ cov}\left[T_{ij},T_{kl}\right]\;S_{kl},$$ (18)

where *S*_*ji*_ and *S*_*kl*_ are the local sensitivity matrix elements defined from Eq. (17) and cov[*T*_*ij*_, *T*_*kl*_] is the covariance of *T*_*ij*_ and *T*_*kl*_.

The local sensitivity matrices for each of the 100 stationary distribution elements are plotted in Fig. S3 of the supplementary material. To have a better picture of microstate sensitivity in relation to local sensitivity, Fig. S4 of the supplementary material plots the element values of the sensitivity matrix for several observables of a preliminary MSM built from short MD trajectories of 1 *µ*s simulation time. We can immediately see a trend where the same few microstates contribute toward the most error in all of the observables. Figure S5 of the supplementary material shows the global sensitivity of the stationary distribution observable, illustrating how the same several microstates have significantly higher sensitivity.

**FIG. 12.**
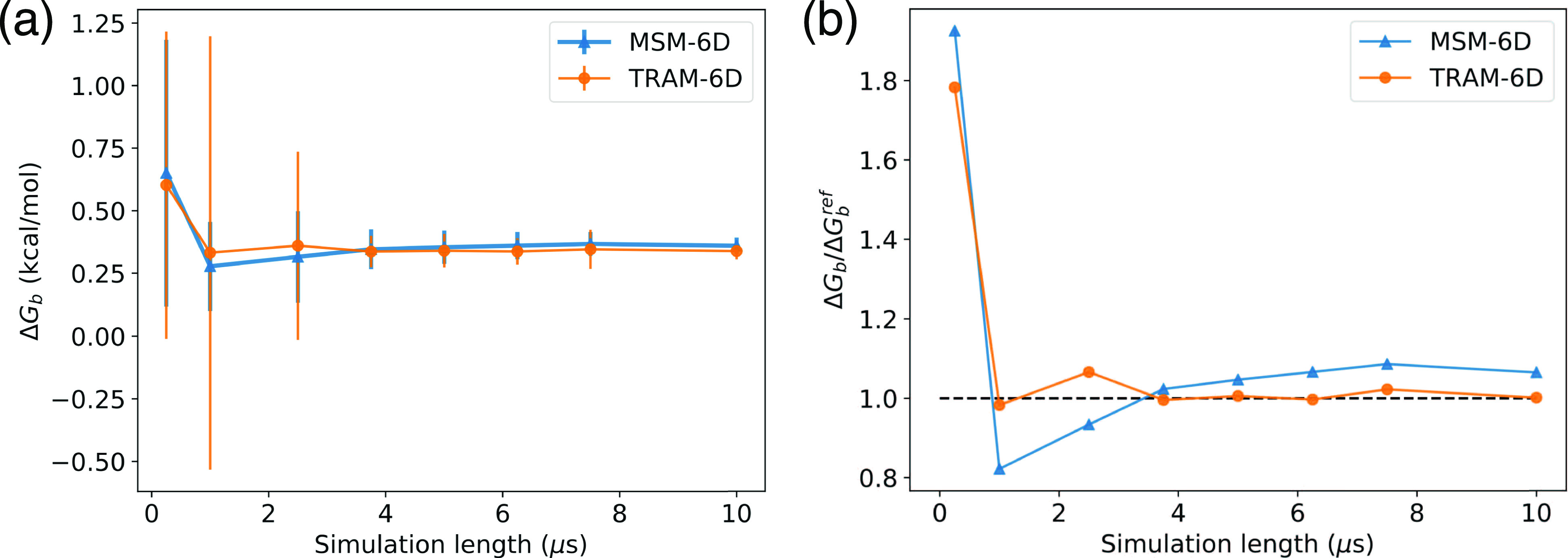
Comparison of MSM and TRAM in six dimensions. (a) Binding free energy Δ*G*_b_ from MSM and TRAM as a function of the MD simulation time. Blocks of trajectories used in the estimation are taken from the 25 *µ*s aggregate MD simulations. For TRAM, the input also included the US trajectories of 200 ns per window with 14 windows biased along a COM distance of 13 Å–26 Å. These 14 US windows were identified by the sensitivity analysis to be the most important windows out of 70 total windows for addition into TRAM. (b) Convergence of Δ*G*_b_ compared to the reference value from MSM-6D built using the full 25 *µ*s of simulation time. After 3.75 *µ*s, TRAM has converged with an error of 18.6%, while MSM has not with a relatively larger error of 23.1%.

Figure 12 compares the binding free energy, Δ*G*_b_, from a six-dimensional traditional MSM and a six-dimensional traditional TRAM over a range of simulation time lengths. The MSM was constructed using the 25 *µ*s aggregate MD simulations. The sensitivity analysis identified 14 US windows out of 70 total windows to be the most important for addition into TRAM, allowing for a much more computationally efficient estimation. Therefore, the following inputs were used to perform reweighting with TRAM: (1) the 25 *µ*s aggregate MD simulations and (2) the US simulations of 200 ns per window with 14 windows biased along a COM distance of 13 Å–26 Å. Bootstrapping was performed to estimate the observable errors and to obtain the error bars in Fig. 12. The Δ*G*_b_ of the MSM fluctuates wildly when we have shorter trajectoriesand only stabilizes when we have 4 *µ*s of aggregate simulation time. In contrast, TRAM starts to generate much more consistent results after only 1 *µ*s of simulation time. While the error bars are relatively large for both the MSM and TRAM estimated with shorter trajectory blocks, which is also observed, in general, for MSMs of full atomistic simulations,^15^ it is encouraging to observe that the average Δ*G*_b_ converges considerably faster to the reference value for TRAM than for the MSM, and TRAM also generally shows lower error. As shown in Fig. 12(b), TRAM converges to the reference value after 3.75 *µ*s with an error of 18.6%, whereas the MSM still has not fully converged with an error of 23.1%. It is interesting to note that when less than 3 *µ*s of MD simulation time was used to build the MSM/TRAM, we observe very high error. This can be explained due to the presence of absorbing states, of which TRAM is also susceptible. When such an absorbing state is reached, the state cannot be left within the timescale of the short simulation. Figure S6 of the supplementary material highlights this absorbing state case: for very short trajectories, we may see little to no transition events to build a reasonable MSM. The properties calculated for TRAM-6D in Table III are taken from the TRAM model built upon the full 25 *µ*s of MD simulations and 200 ns per window of US simulations.

These results show that the presented protocol combining the adaptive TRAM scheme with the sensitivity analysis can further facilitate the construction of more accurate models while reducing the computational cost by potentially orders of magnitudes. By helping to achieve accurate statistical sampling while keeping the magnitude of the computational effort under control, this focused TRAM approach should be especially advantageous in the construction of MSMs for large biomolecular systems.

## IV. CONCLUSION

To obtain accurate thermodynamics and kinetic properties of large systems such as biomolecules and protein complexes, MSMs are generally constructed and then judged on the basis of on dynamical criteria. However, the overall construction process may become inefficient and even ambiguous when the wide range of possibilities for the numerous methodological aspects of MSMs (featurization, discretization, and lag-time) are further compounded by sampling limitations.

In this work, we seek to illustrate the conditions under which MSMs may be able to perform consistently by exploring how models constructed from different features and simulations at different thermodynamic states are able to capture the binding process and produce observables in good agreement with each other. While simulation data from an all-atom model would yield more accurate and realistic information, full atomistic simulations of large biosystems are highly demanding. In this work, we constructed the MSMs based on the CG representation of the barnase–barstar protein complex, where the simplified nature of the model allows for efficiently constructing and evaluating many different MSMs under different conditions.

Taking advantage of the sensitivity analysis, we showed that one can pinpoint precisely where to add these biased simulations with the help of TRAM in order to improve sampling and reduce computational effort. For large protein complexes requiring extensive computations, the ability to incorporate a small set of well-chosen biased simulations in MSMs is expected to be of tremendous value. The next step will be to implement the MSM-based strategy proposed here to process and analyze the results of an all-atom simulation of the barstar–barnase complex with explicit solvent. Using similar features such as the C*α*’s to create a bottom-up CG model, the strategy is expected to impart a more accurate MSM estimation at lower computational costs. This work is underway.

We conclude by noting that the MSMs in this work are quite robust and seem to be invariant to the complexity of input data, even when the number of features is highly reduced. From our CG protein complex, the MSMs are able to resolve the binding process very well. Notably, stripping the features down to one dimension, as discussed in Sec. III B 1, does not seem to affect the MSM, and the observables remain in agreement to those from higher dimensional MSMs. This could prove to be especially applicable for large proteins, of which we could build CG designs and construct their MSMs from a minimal set of features. Given the success of CG modeling for biomolecules,^50–52^ this work serves as a stepping stone for introducing MSM analysis to more rigorously designed reduced models of interest in order to not only accurately but also efficiently reproduce dynamics. Future work includes qualitatively recapitulating the thermodynamic and kinetic information from the all-atomistic barnase–barstar complex, with a special focus on improving the description of the dissociation process through TRAM. For other more complicated protein–protein interactions, Hamiltonian replica exchange molecular dynamics (H-REMD)^53^ may be useful to circumvent sampling issues and aid in the efficiency of TRAM.^54^

One end goal of computational biochemistry is to provide accurate free energies that are comparable with the experimental observables, with errors in the order of *k*_B_*T*. While results are certainly limited by force field accuracy, sampling remains problematic due to the enormous computational costs. The strategies presented here can provide insight for the design of more effective all-atom computations to help overcome sampling challenges, progressively moving toward quantitatively reliable computational predictions.

## SUPPLEMENTARY MATERIAL

See the supplementary material (Table S1 and Figs. S1–S6) for additional information about the analysis.

## Data Availability

The data that support the findings of this study are available from the corresponding author upon request.

## References

[1] W. L. Jorgensen, “The many roles of computation in drug discovery,” Science 303(5665), 1813–1818 (2004). 10.1126/science.1096361 15031495

[2] O. Keskin, N. Tuncbag, and A. Gursoy, “Predicting protein–protein interactions from the molecular to the proteome level,” Chem. Rev. 116(8), 4884–4909 (2016). 10.1021/acs.chemrev.5b00683 27074302

[3] D. E. Scott, A. R. Bayly, C. Abell, and J. Skidmore, “Small molecules, big targets: Drug discovery faces the protein–protein interaction challenge,” Nat. Rev. Drug Discovery 15(8), 533 (2016). 10.1038/nrd.2016.29 27050677

[4] D. Guo, T. Mulder-Krieger, A. P. IJzerman, and L. H. Heitman, “Functional efficacy of adenosine A_2_A receptor agonists is positively correlated to their receptor residence time,” Br. J. Pharmacol. 166(6), 1846–1859 (2012). 10.1111/j.1476-5381.2012.01897.x 22324512 PMC3402809

[5] A. Valencia and F. Pazos, “Computational methods for the prediction of protein interactions,” Curr. Opin. Struct. Biol. 12(3), 368–373 (2002). 10.1016/s0959-440x(02)00333-0 12127457

[6] G. R. Bowman, V. S. Pande, and F. Noé, “Introduction and overview of this book,” in An Introduction to Markov State Models and Their Application to Long Timescale Molecular Simulation (Springer, 2014), pp. 1–6.

[7] J.-H. Prinz, H. Wu, M. Sarich, B. Keller, M. Senne, M. Held, J. D. Chodera, C. Schütte, and F. Noé, “Markov models of molecular kinetics: Generation and validation,” J. Chem. Phys. 134(17), 174105 (2011). 10.1063/1.3565032 21548671

[8] V. S. Pande, K. Beauchamp, and G. R. Bowman, “Everything you wanted to know about Markov state models but were afraid to ask,” Methods 52(1), 99–105 (2010). 10.1016/j.ymeth.2010.06.002 20570730 PMC2933958

[9] M. P. Harrigan, M. M. Sultan, C. X. Hernández, B. E. Husic, P. Eastman, C. R. Schwantes, K. A. Beauchamp, R. T. McGibbon, and V. S. Pande, “MSMBuilder: Statistical models for biomolecular dynamics,” Biophys. J. 112(1), 10–15 (2017). 10.1016/j.bpj.2016.10.042 28076801 PMC5232355

[10] I. Buch, T. Giorgino, and G. De Fabritiis, “Complete reconstruction of an enzyme-inhibitor binding process by molecular dynamics simulations,” Proc. Natl. Acad. Sci. U. S. A. 108(25), 10184–10189 (2011). 10.1073/pnas.1103547108 21646537 PMC3121846

[11] N. Plattner and F. Noé, “Protein conformational plasticity and complex ligand-binding kinetics explored by atomistic simulations and Markov models,” Nat. Commun. 6, 7653 (2015). 10.1038/ncomms8653 26134632 PMC4506540

[12] S. A. Adcock and J. A. McCammon, “Molecular dynamics: Survey of methods for simulating the activity of proteins,” Chem. Rev. 106(5), 1589–1615 (2006). 10.1021/cr040426m 16683746 PMC2547409

[13] G. Pérez-Hernández, F. Paul, T. Giorgino, G. De Fabritiis, and F. Noé, “Identification of slow molecular order parameters for Markov model construction,” J. Chem. Phys. 139(1), 015102 (2013). 10.1063/1.4811489 23822324

[14] C. R. Schwantes and V. S. Pande, “Improvements in Markov state model construction reveal many non-native interactions in the folding of NTL9,” J. Chem. Theory Comput. 9(4), 2000–2009 (2013). 10.1021/ct300878a 23750122 PMC3673732

[15] N. Plattner, S. Doerr, G. De Fabritiis, and F. Noé, “Complete protein–protein association kinetics in atomic detail revealed by molecular dynamics simulations and Markov modelling,” Nat. Chem. 9(10), 1005 (2017). 10.1038/nchem.2785 28937668

[16] A. C. Pan, D. Jacobson, K. Yatsenko, D. Sritharan, T. M. Weinreich, and D. E. Shaw, “Atomic-level characterization of protein–protein association,” Proc. Natl. Acad. Sci. U. S. A. 116(10), 4244–4249 (2019). 10.1073/pnas.1815431116 30760596 PMC6410769

[17] H. Wu, F. Paul, C. Wehmeyer, and F. Noé, “Multiensemble Markov models of molecular thermodynamics and kinetics,” Proc. Natl. Acad. Sci. U. S. A. 113(23), E3221–E3230 (2016). 10.1073/pnas.1525092113 27226302 PMC4988570

[18] D. P. Loucks and E. van Beek, “System sensitivity and uncertainty analysis,” in Water Resource Systems Planning and Management (Springer, 2017), pp. 331–374.

[19] A. Saltelli, M. Ratto, T. Andres, F. Campolongo, J. Cariboni, D. Gatelli, M. Saisana, and S. Tarantola, Global Sensitivity Analysis: The Primer (John Wiley & Sons, 2008).

[20] A. Saltelli, P. Annoni, I. Azzini, F. Campolongo, M. Ratto, and S. Tarantola, “Variance based sensitivity analysis of model output. Design and estimator for the total sensitivity index,” Comput. Phys. Commun. 181(2), 259–270 (2010). 10.1016/j.cpc.2009.09.018

[21] I. M. Sobol, “Sensitivity analysis for non-linear mathematical models,” Math. Modell. Comput. Exp. 1, 407–414 (1993).

[22] N. Singhal and V. S. Pande, “Error analysis and efficient sampling in Markovian state models for molecular dynamics,” J. Chem. Phys. 123(20), 204909 (2005). 10.1063/1.2116947 16351319

[23] G. R. Bowman, K. A. Beauchamp, G. Boxer, and V. S. Pande, “Progress and challenges in the automated construction of Markov state models for full protein systems,” J. Chem. Phys. 131(12), 124101 (2009). 10.1063/1.3216567 19791846 PMC2766407

[24] J. K. Weber and V. S. Pande, “Characterization and rapid sampling of protein folding Markov state model topologies,” J. Chem. Theory Comput. 7(10), 3405–3411 (2011). 10.1021/ct2004484 22140370 PMC3226725

[25] A. M. Buckle, G. Schreiber, and A. R. Fersht, “Protein-protein recognition: Crystal structural analysis of a barnase-barstar complex at 2.0-.ANG. resolution,” Biochemistry 33(30), 8878–8889 (1994). 10.1021/bi00196a004 8043575

[26] R. D. Hills and C. L. Brooks, “Insights from coarse-grained Gō models for protein folding and dynamics,” Int. J. Mol. Sci. 10(3), 889–905 (2009). 10.3390/ijms10030889 19399227 PMC2672008

[27] S. Takada, “Coarse-grained molecular simulations of large biomolecules,” Curr. Opin. Struct. Biol. 22(2), 130–137 (2012). 10.1016/j.sbi.2012.01.010 22365574

[28] G. Schreiber and A. R. Fersht, “Interaction of barnase with its polypeptide inhibitor barstar studied by protein engineering,” Biochemistry 32(19), 5145–5150 (1993). 10.1021/bi00070a025 8494892

[29] S. Wu, C. Jun Lee, and L. G. Pedersen, “Analysis on long-range residue–residue communication using molecular dynamics,” Proteins: Struct., Funct., Bioinf. 82(11), 2896–2901 (2014). 10.1002/prot.24629 PMC4206573 24935629

[30] H. B. L. Jones, S. A. Wells, E. J. Prentice, A. Kwok, L. L. Liang, V. L. Arcus, and C. R. Pudney, “A complete thermodynamic analysis of enzyme turnover links the free energy landscape to enzyme catalysis,” FEBS J. 284(17), 2829–2842 (2017). 10.1111/febs.14152 28650586

[31] J. C. Phillips, R. Braun, W. Wang, J. Gumbart, E. Tajkhorshid, E. Villa, C. Chipot, R. D. Skeel, L. Kalé, and K. Schulten, “Scalable molecular dynamics with NAMD,” J. Comput. Chem. 26(16), 1781–1802 (2005). 10.1002/jcc.20289 16222654 PMC2486339

[32] B. R. Brooks, C. L. Brooks III, A. D. Mackerell, Jr., L. Nilsson, R. J. Petrella, B. Roux, Y. Won, G. Archontis, C. Bartels, S. Boresch *et al.*, “CHARMM: The biomolecular simulation program,” J. Comput. Chem. 30(10), 1545–1614 (2009). 10.1002/jcc.21287 19444816 PMC2810661

[33] W. Humphrey, A. Dalke, and K. Schulten, “VMD: Visual molecular dynamics,” J. Mol. Graphics 14, 33–38 (1996). 10.1016/0263-7855(96)00018-5 8744570

[34] H. Steinhaus, “Sur la division des corp materiels en parties,” Bull. Acad. Polon. Sci. IV(C1.III), 801–804 (1956).

[35] J. MacQueen, “Some methods for classification and analysis of multivariate observations,” in *Proceedings of the Fifth Berkeley Symposium on Mathematical Statistics and Probability* (University of California Press, 1967), Vol. 1, pp. 281–297.

[36] R. L. Thorndike, “Who belongs in the family?,” Psychometrika 18(4), 267–276 (1953). 10.1007/bf02289263

[37] R. T. McGibbon, K. A. Beauchamp, M. P. Harrigan, C. Klein, J. M. Swails, C. X. Hernández, C. R. Schwantes, L.-P. Wang, T. J. Lane, and V. S. Pande, “MDTraj: A modern open library for the analysis of molecular dynamics trajectories,” Biophys. J. 109(8), 1528–1532 (2015). 10.1016/j.bpj.2015.08.015 26488642 PMC4623899

[38] M. K. Scherer, B. Trendelkamp-Schroer, F. Paul, G. Pérez-Hernández, M. Hoffmann, N. Plattner, C. Wehmeyer, J.-H. Prinz, and F. Noé, “PyEMMA 2: A software package for estimation, validation, and analysis of Markov models,” J. Chem. Theory Comput. 11(11), 5525–5542 (2015). 10.1021/acs.jctc.5b00743 26574340

[39] J. D. Hunter, “Matplotlib: A 2D graphics environment,” Comput. Sci. Eng. 9(3), 90–95 (2007). 10.1109/MCSE.2007.55

[40] R. T. McGibbon and V. S. Pande, “Variational cross-validation of slow dynamical modes in molecular kinetics,” J. Chem. Phys. 142(12), 124105 (2015). 10.1063/1.4916292 25833563 PMC4398134

[41] B. E. Husic, R. T. McGibbon, M. M. Sultan, and V. S. Pande, “Optimized parameter selection reveals trends in Markov state models for protein folding,” J. Chem. Phys. 145(19), 194103 (2016). 10.1063/1.4967809 27875868 PMC5116026

[42] C. Zhao and D. Shukla, “Structural basis for negative regulation of ABA signaling by ROP11 GTPase,” 10.1101/2020.05.20.107185 (2020). 10.1101/2020.05.20.107185 40966032

[43] D. Meral, D. Provasi, D. Prada-Gracia, J. Möller, K. Marino, M. J. Lohse, and M. Filizola, “Molecular details of dimerization kinetics reveal negligible populations of transient *μ*-opioid receptor homodimers at physiological concentrations,” Sci. Rep. 8(1), 7705 (2018). 10.1038/s41598-018-26070-8 29769636 PMC5955887

[44] C. Wehmeyer, M. K. Scherer, T. Hempel, B. E. Husic, S. Olsson, and F. Noé, “Introduction to Markov state modeling with the PyEMMA software [Article v1.0],” Living J. Comput. Mol. Sci. 1(1), 5965 (2019). 10.33011/livecoms.1.1.5965

[45] H. Wu and F. Noé, “Variational approach for learning Markov processes from time series data,” J. Nonlinear Sci. 30, 23–66 (2020). 10.1007/s00332-019-09567-y

[46] L. Wang, S. W. I. Siu, W. Gu, and V. Helms, “Downhill binding energy surface of the barnase–barstar complex,” Biopolymers 93(11), 977–985 (2010). 10.1002/bip.21507 20540151

[47] S. Röblitz and M. Weber, “Fuzzy spectral clustering by PCCA+: Application to Markov state models and data classification,” Adv. Data Anal. Classif. 7(2), 147–179 (2013). 10.1007/s11634-013-0134-6

[48] F. Noé, H. Wu, J.-H. Prinz, and N. Plattner, “Projected and hidden Markov models for calculating kinetics and metastable states of complex molecules,” J. Chem. Phys. 139(18), 184114 (2013). 10.1063/1.4828816 24320261

[49] I. M. Sobol, “Global sensitivity indices for nonlinear mathematical models and their Monte Carlo estimates,” Math. Comput. Simul. 55(1-3), 271–280 (2001). 10.1016/S0378-4754(00)00270-6

[50] W. G. Noid, “Perspective: Coarse-grained models for biomolecular systems,” J. Chem. Phys. 139(9), 090901 (2013). 10.1063/1.4818908 24028092

[51] M. Baaden and S. J. Marrink, “Coarse-grain modelling of protein–protein interactions,” Curr. Opin. Struct. Biol. 23(6), 878–886 (2013). 10.1016/j.sbi.2013.09.004 24172141

[52] M. G. Saunders and G. A. Voth, “Coarse-graining methods for computational biology,” Annu. Rev. Biophys. 42, 73–93 (2013). 10.1146/annurev-biophys-083012-130348 23451897

[53] Y. Sugita and Y. Okamoto, “Replica-exchange multicanonical algorithm and multicanonical replica-exchange method for simulating systems with rough energy landscape,” Chem. Phys. Lett. 329(3-4), 261–270 (2000). 10.1016/s0009-2614(00)00999-4

[54] F. Paul, C. Wehmeyer, E. T. Abualrous, H. Wu, M. D. Crabtree, J. Schöneberg, J. Clarke, C. Freund, T. R. Weikl, and F. Noé, “Protein-peptide association kinetics beyond the seconds timescale from atomistic simulations,” Nat. Commun. 8(1), 1095 (2017). 10.1038/s41467-017-01163-6 29062047 PMC5653669

